# International Expert Opinions and Recommendations on the Use of Melatonin in the Treatment of Insomnia and Circadian Sleep Disturbances in Adult Neuropsychiatric Disorders

**DOI:** 10.3389/fpsyt.2021.688890

**Published:** 2021-06-10

**Authors:** Laura Palagini, Raffaele Manni, Eugenio Aguglia, Mario Amore, Roberto Brugnoli, Stéphanie Bioulac, Patrice Bourgin, Jean-Arthur Micoulaud Franchi, Paolo Girardi, Luigi Grassi, Régis Lopez, Claudio Mencacci, Giuseppe Plazzi, Julia Maruani, Antonino Minervino, Pierre Philip, Sylvie Royant Parola, Isabelle Poirot, Lino Nobili, Giovanni Biggio, Carmen M. Schroder, Pierre A. Geoffroy

**Affiliations:** ^1^Psychiatry Division, Department of Clinical and Experimental Medicine, University of Pisa, Pisa, Italy; ^2^Unit of Sleep Medicine and Epilepsy, Istituto di Ricovero e Cura a Carattere Scientifico Mondino Foundation, Pavia, Italy; ^3^Department of Experimental and Clinical Medicine, Psychiatric Clinic University Hospital “Gaspare Rodolico”, University of Catania, Catania, Italy; ^4^Section of Psychiatry, Department of Neuroscience, Rehabilitation, Ophthalmology, Genetics, Maternal and Child Health, University of Genoa, Genoa, Italy; ^5^Istituto di Ricovero e Cura a Carattere Scientifico Ospedale Policlinico San Martino, Genoa, Italy; ^6^Department of Neuroscience, Mental Health, and Sensory Organs (NESMOS), Faculty of Medicine and Psychology, Sant'Andrea University Hospital, Sapienza University, Rome, Italy; ^7^University Sleep Clinic, Services of Functional Exploration of the Nervous System, University Hospital of Bordeaux, and USR CNRS 3413 SANPSY, University Hospital Pellegrin, University of Bordeaux, Bordeaux, France; ^8^Institut des Neurosciences Cellulaires et Intégratives, CNRS-UPR 3212, Strasbourg, France; ^9^Centre des troubles du sommeil - CIRCSom, Strasbourg University Hospitals, Strasbourg, France; ^10^Department of Neuroscience and Rehabilitation, University of Ferrara, Ferrara, Italy; ^11^Service de Neurologie, Centre National de Référence Narcolepsie Hypersomnies, Unité des Troubles du Sommeil, Hôpital Gui-de-Chauliac Montpellier, Montpellier, France; ^12^PSNREC, Univ Montpellier, INSERM, Montpellier, France; ^13^Department of Neuroscience, Aziende Socio Sanitarie Territoriali Fatebenefratelli Sacco, Milan, Italy; ^14^Department of Biomedical, Metabolic and Neural Sciences, University of Modena and Reggio Emilia, Modena, Italy; ^15^Istituto di Ricovero e Cura a Carattere Scientifico, Institute of Neurological Sciences of Bologna, Bologna, Italy; ^16^Département de psychiatrie et d'addictologie, AP-HP, GHU Paris Nord, DMU Neurosciences, Hopital Bichat - Claude Bernard, Paris, France; ^17^Department of Psychiatry, Italian Society of Psychosomatic Medicine (SIMP), Parma, Italy; ^18^Reseau Morphee Paris, Paris, France; ^19^Service de psychiatrie adulte, Hôpital Fontan, CHU de Lille, Lille, France; ^20^Department of Neuroscience (DINOGMI), University of Genoa, Genoa, Italy; ^21^Istituto di Ricovero e Cura a Carattere Scientifico, Child Neuropsychiatry Unit, Giannina Gaslini Institute, Genoa, Italy; ^22^Department of Life and Environmental Sciences, Institute of Neuroscience, CNR, University of Cagliari, Cagliari, Italy; ^23^Department of Child and Adolescent Psychiatry, Strasbourg University Hospitals, Strasbourg, France; ^24^GHU Paris - Psychiatry & Neurosciences, Paris, France; ^25^CNRS UPR 3212, Institute for Cellular and Integrative Neurosciences, Strasbourg, France; ^26^Université de Paris, NeuroDiderot, Inserm, Paris, France

**Keywords:** insomnia, circadian sleep disorders, melatonin, expert opinion, psychiatric disorders, delirium, dementia

## Abstract

**Introduction:** Insomnia and circadian rhythm disorders, such as the delayed sleep phase syndrome, are frequent in psychiatric disorders and their evaluation and management in early stages should be a priority. The aim of this paper was to express recommendations on the use of exogenous melatonin, which exhibits both chronobiotic and sleep-promoting actions, for the treatment of these sleep disturbances in psychiatric disorders.

**Methods:** To this aim, we conducted a systematic review according to PRISMA on the use of melatonin for the treatment of insomnia and circadian sleep disorders in neuropsychiatry. We expressed recommendations for the use of melatonin in psychiatric clinical practice for each disorder using the RAND/UCLA appropriateness method.

**Results:** We selected 41 studies, which included mood disorders, schizophrenia, substance use disorders, attention deficit hyperactivity disorders, autism spectrum disorders, neurocognitive disorders, and delirium; no studies were found for both anxiety and eating disorders.

**Conclusion:** The administration of prolonged release melatonin at 2–10 mg, 1–2 h before bedtime, might be used in the treatment of insomnia symptoms or comorbid insomnia in mood disorders, schizophrenia, in adults with autism spectrum disorders, neurocognitive disorders and during sedative-hypnotics discontinuation. Immediate release melatonin at <1 mg might be useful in the treatment of circadian sleep disturbances of neuropsychiatric disorders.

## Introduction

Insomnia and circadian rhythm disorders are frequent in psychiatric disorders. In particular, insomnia can be a risk factor, as well as a comorbid condition, or a symptom and an early sign of psychiatric disorders (1–4). Insomnia is the most common sleep disturbance associated with psychiatric disorders and may emerge during any stage of illness. It includes prodromal, first episode, acute, recurrence, and even remission stages, thereby being associated with a worse course of illness, increased symptom severity, relapses or recurrences, and increased suicidal risk (1–7). Thus, insomnia symptoms may be an important modifiable risk factor to prevent psychiatric disorders and/or achieve and maintain remission. Thereby insomnia evaluation and management in early stages should be a priority in psychiatric cares. Guidelines for insomnia treatment approved pharmacological options such as benzodiazepines and benzodiazepine receptor agonists (so called Z-drugs) (8, 9). Although both benzodiazepines and Z-drugs have been in use for more than 40 years for the management of insomnia, their adverse side effects discourage the use of these drugs especially over extended periods. Risks of hangover, nocturnal confusion, falls, negative effects on next-day cognitive performance, rebound insomnia, tolerance, and dependency make their use controversial and not without risks (8, 9). In addition, the worsening of mood symptoms including mood swings, suicidal thoughts, and behaviors have been reported in association with the use of sedatives/hypnotics (8, 9). An additional and independent concern is that Z-drugs can cause parasomnias, which in rare cases, may lead to suicidal ideation and behaviors (8, 9). According to recent European guidelines, other compounds, including antidepressants, antihistamines, antipsychotics, or antiepileptics which have been used for decades, should not be considered as first line treatment options (8, 9). Melatonin is a neurohormone synthesized and secreted by the pineal gland at night. Among its many physiological functions, melatonin has both a chronobiotic action such as it entrains the circadian rhythms of several biological functions including sleep/wake rhythms and a soporific effect with a sleep-promoting action (10, 11). In psychiatric disorders, either one of these two actions and their combination could have interesting effects in clinical practice (10, 11). In line with this, guidelines for the treatment of circadian rhythm disorders already underline the role of exogenous melatonin as a first line option (8, 11, 12). Moreover, a recent consensus of the British Association for Psychopharmacology and a recent expert opinions and consensus recommendations of five Italian scientific societies concluded that 2 mg prolonged release melatonin (PRM) (e.g., Circadin®, developed by Neurim, Tel Aviv, Israel) should be the first-choice treatment when a hypnotic is indicated in patients over 55 years. In 2007, the European Medicines Agency (EMA) authorized the use of melatonin (Circadin®) as medication; however, melatonin has also used as a dietary supplement in many European countries. Melatonin is the main hormone involved in the control of the sleep-wake cycle, which decreases with age, and its deficit is at least partly responsible for insomnia symptoms, warranting the use of exogenous melatonin for the treatment of insomnia in the elderly. Melatonin regulation received wide attention across the psychopathological spectrum, and beyond insomnia, patients suffering from neuropsychiatric disorders usually have abnormal amount and timing of melatonin secretions. These alterations in melatonin secretion may be linked to circadian rhythm alterations that are common in many neuropsychiatric disorders including anxiety, depression, bipolar disorder, schizophrenia (11). Alterations in circadian rhythm are considered risk factors for the onset of psychiatric disorders, precursors of relapse, associated with residual symptoms, treatment resistance, and increased suicidal risk. According to the “circadian hypothesis” of psychiatric disorders (13, 14) the de-synchronization of the master biological clock of the hypothalamus, the suprachiasmatic nuclei, constitutes a hallmark and a key feature of psychiatric disorders. In particular, patients with psychiatric disorders and circadian sleep disorders may present polymorphisms of different circadian clock genes and mutations in melatonin receptors, which may alter the biological clock (13, 14). These findings suggested that, as for insomnia, altered circadian rhythms might be important modifiable risk factors to prevent psychiatric disorders and/or achieve and maintain remission. Finally, melatonin can also be used as a treatment for benzodiazepines and hypnotics discontinuation in patients with insomnia (15).

In this context of increasing interest for the use of melatonin in psychiatric disorders, the main aim of this consensus paper is to conduct an overview on the use of melatonin for the treatment of insomnia and circadian sleep problems in psychiatric disorders and to express recommendations on its use in psychiatric clinical practice. PR melatonin at 2 mg is considered pharmacologically active and corresponds to a medicinal dose that can only be delivered on medical prescription, with an indication by the EMA as a monotherapy for insomnia (9, 11). Exogenous melatonin is therefore accessible over the counter in many countries and in many forms: tablets, capsules, orodispersible, oral solution, or spray, however in large majority as an immediate release (IR) formula, and is considered a food supplement at the dose below 2 mg with no dose or age limits (11). The French Society for Sleep Research and Sleep Medicine (SFRMS), aware of the issues linked to an increasing consumption of melatonin and aiming to optimize its therapeutic use, appointed already in 2018 a group of experts who worked in a consensus conference on melatonin prescription in psychiatric disorders (11). The present recommendations are the result of an international task force based on the French and Italian experiences, with an expertise in the field of sleep medicine, psychosomatic medicine, psychiatric practice, neuropsychopharmacology, and psychopharmacology for optimizing therapeutic use of melatonin in insomnia and circadian sleep disorders in the psychiatric clinical practice.

## Methods

The Italian Society of Psychosomatic Medicine (SIMP) promoted and commissioned the present international expert consensus recommendations, with the Italian Association of Sleep Medicine (AIMS), the Italian Association for the Fight Against Stigma (AILAS), the Italian Society of Consultation-Liaison Psychiatry (SIPC), the Italian Society of Neuropsychopharmacology (SINF), and the Italian Society of Psychosomatic Medicine (SIMP) participating to this project. Both the French Society for Sleep Research and Sleep Medicine (SFRMS), and the French Association of Biological Psychiatry and Neuropsychopharmacology (AFPBN) contributed to these guidelines. The development process was based on the RAND/UCLA Appropriateness Method (16) for conceptualizing, designing, and carrying out the appropriateness of procedures for the use of melatonin in the treatment of insomnia and circadian sleep disorders in psychiatric disturbances. The method consists of a modified Delphi approach (17) in which a panel of Italian and French experts assessed the appropriateness of particular clinical decisions to clinical psychiatric practice in an iterative way. Initially, a literature review and synthesis was conducted to critically appraise and summarize the evidence (LP, PAG). The literature review was carried out up to November 2020 on the use of melatonin in the treatment of insomnia and circadian sleep disorders in the following adult psychiatric disorders: bipolar disorders, major depressive disorders, seasonal affective disorders, psychotic disorders, anxiety disorders, attention deficit hyperactivity disorders (ADHDs) in adults, autism spectrum disorders (ASD) in adults, substance abuse disorders, eating disorders, delirium, neurocognitive disorders. We decided not to consider melatonin for preoperative and postoperative anxiety which is not a proper psychiatric disorder according to international classifications, nor melatonin agonists such as for example ramelteon which have been indeed summarized in the French consensus (11).

The PubMed, PsycINFO, and Embase electronic databases were searched for literature published according to the PRISMA (Preferred Reporting Items for Systematic reviews and Meta-Analysis) method (18). Several combinations of search terms were used such as “melatonin and insomnia treatment” or “melatonin and circadian sleep disorders treatment” and “bipolar disorders” or “major depressive disorders” or “seasonal affective disorders” or “psychotic disorders” or “anxiety disorder” or “adult ADHDs” or “adult ASD” or “eating disorders” or “substance abuse disorders” or “delirium” or “neurocognitive disorders” were included. Inclusion criteria were (1) adult population; (2) full text available in English; (3) performed up to November 2020. Systematic reviews and meta analyses were included while papers were excluded if they concerned other sleep disorders or were related to sleep disturbances in general, were conducted in special populations or in children and adolescents with special needs.

The panel of experts in the field was then asked to rate the appropriateness of the recommendations selected according to the Delphi approach (17). The Panel experts were asked to rate recommendations in two rounds (1rst round with no interaction, 2nd round during a web discussion) and to express the extent of appropriateness using a 9-point scale in which 9 = extremely appropriate, 1 = extremely inappropriate, and 5 = equivocal or uncertain. Based on the median score and the extent of agreement for each of the indications a panel statement was calculated and those statements not achieving consensus (median scores <7) were not inserted in the paper or were reformulated. The final Consensus Recommendation Statement resulted from the third round of voting. The first part of the paper summarized the main characteristics of melatonin as a neurohormone and of its alterations in insomnia, circadian sleep disorders and psychiatric disorders. In this part, a brief overview on exogenous melatonin drugs currently available on the market and commonly used in the clinical practice has been conducted. The second part summarized the main findings on the use of melatonin for the treatment of insomnia and circadian rhythm disorders in each psychiatric disorders examined and then included in the main Recommendation Statements.

## Results

**Figure 1:**
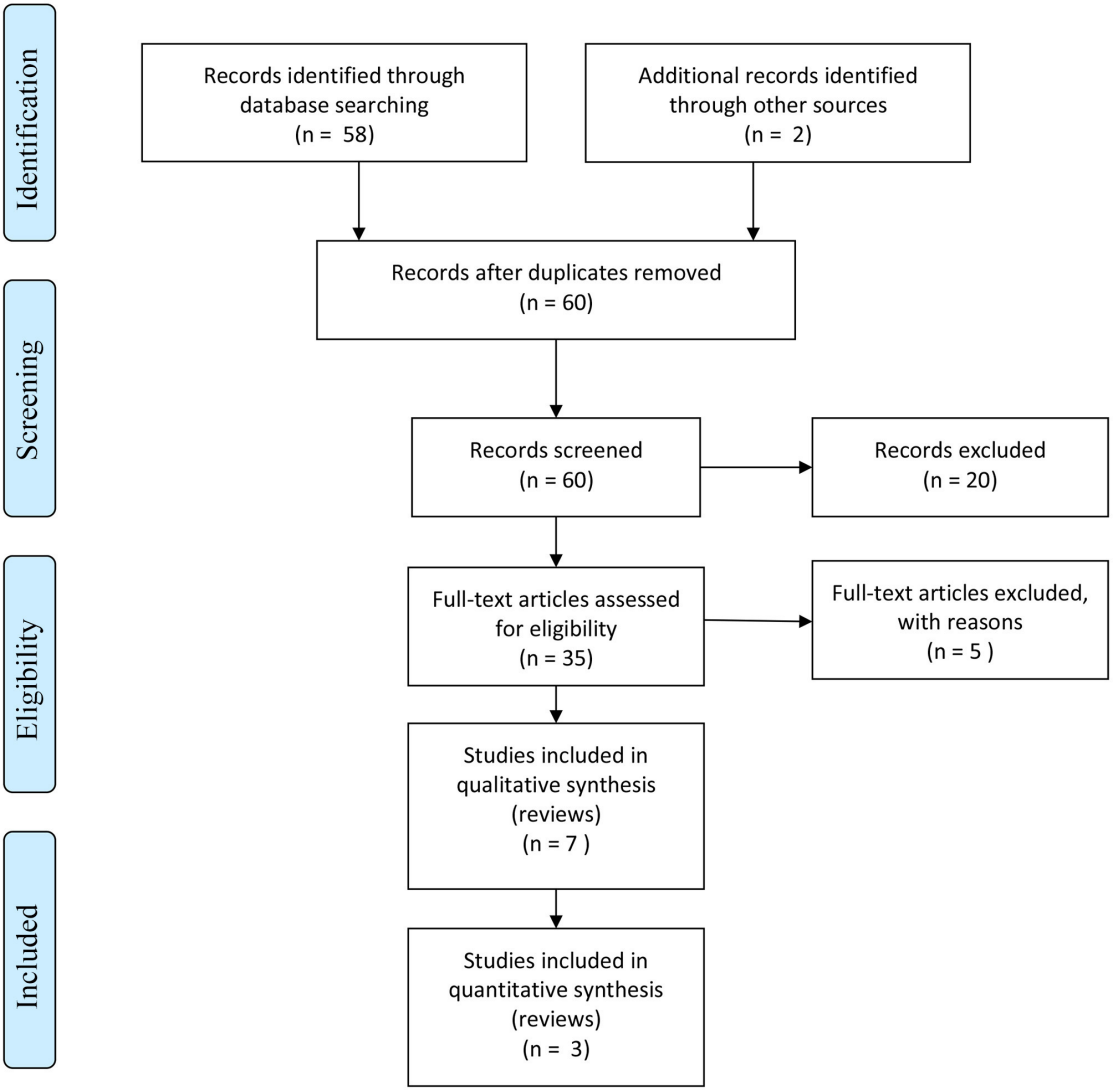
PRISMA flow diagram.

Based on this search, 60 articles were identified, with 35 articles being selected according to the inclusion/exclusion criteria, including most recent meta-analyses and systematic reviews. Thirteen articles were considered for mood disorders, 1 for ADHD in adults, 1 for ASD in adults, 5 for delirium, 7 for neurocognitive disorders, 6 for schizophrenia, 2 for substance use disorders, and no studies for anxiety and eating disorders (Figure 1). The resulting recommendations by the international expert task force regarding potential indications of melatonin for insomnia and circadian rhythm disorders in adult with psychiatric disorders are summarized after each disorder taken into consideration.

### Overview on Melatonin and on Currently Available Exogenous Melatonin Formula

Melatonin (N-acetyl-5-methoxytryptamine) known as the hormone of the “darkness,” is a peptide hormone synthesized and secreted by the pineal gland in the brain and directly released in the blood. Production is induced by signals from the internal biological clock localized in the suprachiasmatic nuclei (SCN) of the hypothalamus and is inhibited by light perceived by the retina (11, 13, 19). Light stimulus, in particular in the blue range, activates melanopsin breakdown in retinal photoreceptive ganglion cells that project to the hypothalamus and inhibit melatonin synthesis. Melatonin developed several mechanisms of actions and acts on its own receptors (MT1 and MT2), which are members of the G-protein-linked receptor family. Their activation leads to the inhibition of the adenylate cyclase which, in turn, provokes the inhibition of protein kinase A and of CREB protein phosphorylation (11, 13, 19). Melatonin thus controls transcription action of clock genes that are part of a complex molecular machinery resulting in proteins, which control throughout the 24-h cycle, the transcription and translation of other clock-controlled genes. The precise daily repetition of melatonin signal and its relation to the dark phase of the day makes melatonin the internal synchronizer of circadian rhythms and defines the so-called “chronobiotic action” of melatonin (11, 13, 19). Even though melatonin is a neurohormone, it is important to note that its secretion is not submitted to a classical negative feedback control, but to an original mechanism at the level of the SCN which in turn contributes to modify its own secretion phase (11, 13, 19).

As such, when given as a treatment, exogenous melatonin acts as an external synchronizer of the circadian rhythm and induces a phase advance or phase delay of its own secretion depending on the timing of its administration. Overall, melatonin is one of the most powerful synchronizers of human circadian biological rhythms and acts as a major chronobiological pacemaker. As such, the pineal gland and the SNC act together in order to synchronize peripheral clocks and melatonin is involved in signaling the “time of day” and “time of year” (11, 13, 19). Melatonin has also many other actions, such as antioxidant, antiapoptotic, immune-enhancing, and oncostatic properties [for an overview see (11, 13, 19, 20)]. Melatonin hormonal dysfunction includes decreased melatonin nocturnal peak production and melatonin circadian displacement. The production of melatonin generally decreases with age and it is related to an increase in insomnia prevalence in the elderly. This relationship between increasing age, declining melatonin production and increasing insomnia prevalence has led to the “melatonin-replacement” hypothesis. In this framework, treatments with melatonin may replenish the deficiency in the endogenous hormone involved in sleep promotion and may overall improve sleep quality (21).

Nerveless, a decreased level of melatonin has been described even in young adults with insomnia (22). Changes in melatonin secretion have been observed since the eighties in bipolar disorders, with a delayed peak in nocturnal secretion, decreased amplitude of secretion and a greater sensitivity to suppression with light. Alterations of circadian rhythm in major depression, particularly unipolar depression include lower nocturnal melatonin levels, phase advance of melatonin onset or peak, a delay in the peak, onset, or offset of melatonin secretion (11, 13, 19, 20). In 1976, the “low melatonin syndrome” was proposed. Although evidence is somewhat inconsistent, these alterations of melatonin secretions were observed during acute depressive phase, remitted phases, and manic phases, leading to consider them as trait markers of bipolar disorder (23). Melatonin circadian displacement is a hallmark of circadian sleep disorders with a misalignment between the sleep period and the physical/social 24-h environmental cycle. Patients with mood disorders have repeatedly been shown to hold instable circadian rhythms when compared to healthy control subjects (11, 13, 19, 20). This has been related to alteration in melatonin kinetics, as several studies observed that patients with mood disorders have abnormal melatonin secretion at night, later nocturnal peak secretion, reduced secretion amplitude, and a super-sensitive suppression of the melatonin secretion to light (11, 13, 19, 20). The theory that disturbances of sleep and mood have a shared pathophysiology is not new. It has been postulated that sleep problems, circadian rhythm disruption, and mood disturbances are either fundamental responses of a shared common mechanism, or can occur reciprocally (11, 13, 19, 20). A common pathophysiological overlap between circadian rhythm disruption and mood disorders has appeared: many of the same features of circadian rhythm sleep disorders can be seen in mood disorders, such as delayed sleep onset and early morning awakening as well as reversal of the normal peaks of energy, mood, and alertness. The role of melatonin in the seasonal changes in physiology and behavior has been extensively documented (24) and the melatonin rhythm is delayed during winter compared with the summer (24). Changes in duration and/or phase of melatonin secretion during this period, the so called “phase shift hypothesis,” were hypothesized to play a role in the pathogenesis of Seasonal Affective Disorder (SAD) and these studies promoted its treatment with light therapy. In patients with SAD, the most prominent marker of seasonal variations may be represented by the change in the melatonin. In fact, several studies have documented higher daytime melatonin levels during the winter season in patients affected with SAD compared to healthy controls and phase delay of circadian rhythms during the winter (24). Nevertheless, melatonin circadian displacement with irregular rhythms or delayed sleep phase secretion characterize also anxiety disorders, schizophrenia, adults with ADHD and ASD, and patients with eating disorders (25). Sleep and circadian rhythm disorders have also been well-defined in a number of substances use disorders, including those of cannabis, alcohol, nicotine, benzodiazepines, and cocaine, of neurocognitive disorders such as Alzheimer Disease and Parkinson Disease and delirium (19, 26–33). Patients with these psychiatric disorders may present polymorphisms of different circadian clock genes and mutations in melatonin pathway-related genes and receptors which may contribute to alterations in melatonin kinetics (13, 14).

In this context, the exogeneous administration of melatonin may help to stabilize mood, as well as sleep-wake cycle disorders, and prevent psychiatric relapse and recurrence. When given exogenously, melatonin possesses the same sleep-promoting as well as the phase-shifting effects of melatonin. These effects are thought to be mediated by melatonin receptors in the SCN. The role of melatonin in the control of sleep has been investigated in both diurnal and nocturnal species. Local injection of pharmacological amounts of melatonin (1–50 μg) in the medial preoptic area of the rat hypothalamus increased sleep duration in a dose-dependent manner, mainly by increasing non-rapid eye movement sleep (34). Melatonin has been shown to induce sleep by altering the functions of the GABA_A_-benzodiazepine receptor complex. The suppression of neuronal activity by melatonin is one of the possible mechanisms by which this hormone contributes to the regulation of sleep (30, 34). Exogenous melatonin also influences the functioning of the biological clock SCN which responds by an advance or delay, according to the time of administration hence showing a chronobiotic effect. The chronobiotic effect of exogenous melatonin is due to the presence of MT1 and MT2 receptors in the SCN. MT1 receptors inhibit multi-unit activity, while MT2 receptors are responsible for phase shifting responses (11, 19). Hence exogenous melatonin has many properties with two effects on sleep-wake cycles: a sleep-promoting effect called “soporific” and a synchronization effect called “chronobiotic” (11, 19).

Melatonin pharmacokinetics depend on the way of its administration such as oral, immediate Release-IR and/or Prolonged Release-PR, intravenous, nasal spray, anal suppository, skin patches or cream, and on the individual absorption and hepatic metabolization (35). Usually, in a young/middle-aged human patient, pharmacokinetic studies show that plasma concentration reaches the peak at approximately between 20 min and 2 h. Fast-release melatonin preparations have showed inconstant effects in insomnia (36). Melatonin displays a short blood half-life, a fast turn over and undergoes a high first-pass hepatic metabolism. More than 80% is excreted exclusively in the urine as 6-sulfatoxymelatonin. The individual's capacity to produce the endogenous hormone, the decline in circadian clock output and the increase in complaints of poor sleep quality at older age led to the development a prolonged-release melatonin preparation to mimic the endogenous secretion in patients such as PR-melatonin, PRM or Circadin® (Neurim Pharma-ceuticals, Tel-Aviv, Israel). This formulation results in peak of plasma levels 2.6 h after ingestion, which are maintained for 3.5 h. This formulation has a sleep onset such as soporific effect and sleep maintenance promoting effect and is approved and recommended for the treatment of insomnia in subjects over 55 years of age. PRM 2 mg was shown to mimic the physiological release of melatonin by releasing melatonin gradually and acting on melatonin receptors. It has been shown to be effective in improving sleep onset latency, wake time after sleep onset, total sleep time, sleep efficiency, and to reduce night awakenings without altering the physiological sleep architecture. PRM 2 mg has been proven to be well-tolerated and not associated with impairment of psychomotor functions, memory recall, driving skills and has not shown side-effects such as hangover, nocturnal confusion and falls, negative effects on next-day cognitive performance, rebound insomnia, tolerance, or dependency (8, 12, 36–38). Exogenous melatonin is usually remarkably well-tolerated and has overall a high safety profile (30).

Regarding Italy and France, the PRM formulation (Circadin® at 2 mg) has been approved for the treatment of chronic insomnia characterized by poor quality of sleep in people aged 55 years or older, and it is available on the market with medical prescription (9, 11). The maximum concentration allowed for melatonin based food supplements is 1 mg in Italy and up to 1.9 mg in France, in immediate release formulation, with lack of evidence regarding efficacy and safety of the many different commercial products available for insomnia symptoms (39).

Melatonin's “chronobiotic” action exists even at low doses from 0.125 mg and is conserved with increasing dose. Exogenous melatonin can thus be considered a “chronobiotic substance” since it can change the characteristics of a rhythm period, amplitude or phase. Indeed, exogeneous melatonin as well as endogenous melatonin, according to the time of administration, can advance or delay the functioning of the biological clock (11, 19). A biological rhythms phase advance occurs when melatonin is administered in the afternoon with a maximal effect 4–5 h before the start of the endogenous melatonin secretion (11, 19). Guidelines on delayed sleep phase disorder (DSPS) recommend that melatonin should be administrated between 1.5 and 6.5 h prior to the Dim Light Melatonin Onset (DLMO) for 4 weeks in DSPS patients; the DLMO corresponds to the rise of melatonin under physiological conditions in dim light i.e., normally between 7:30 p.m. and 9:30 p.m. in adults with intermediate circadian phases. On the other hand, a biological rhythms phase delay occurs when melatonin is administered in the morning (11, 19).

### Use of Exogenous Melatonin in Mood Disorders: Review of Evidence

In 1997, the Leibenluft team carried out a randomized crossover double-blind trial assessing the use of 10 mg of IR melatonin administered at 10 p.m. over a 12-weeks period to five patients with bipolar disorder, with a placebo and a 1-month free period between the two treatment phases (40). The results did not show any significant benefit of the exogenous administration of IR melatonin on sleep parameters compared to placebo. Bersani and Garavini (41) conducted an open non-controlled trial using 3 mg of IR melatonin to treat insomnia in 11 patients with bipolar disorder and in manic episode for at least 1 month. The study showed efficacy on total sleep time, which was improved by an extra 3 h, and a significant decrease in manic symptoms. Livianos et al. (42) studied a cohort of 14 patients during euthymic phase bipolar disorder and insomnia. Authors observed that IR melatonin at bedtime administered at doses ranging from 3 to 6 mg in combination with the usual treatment, contributed to improving the quality and duration of sleep, with a decrease in residual depressive's symptoms.

Romo-Nava et al. (43) carried out a randomized double-blind study against placebo, assessing 5 mg of PR melatonin over an 8-week period in 20 patients with BD and treated with second-generation antipsychotics. The main objective of this study was to examine metabolic parameters, observing an improvement under melatonin compared to placebo. Regarding manic episodes, McElroy et al. (44) examined the effect at 8 weeks of the melatonin agonist ramelteon 8 mg in 21 patients during manic phases, in combination with an anti-manic treatment. These authors observed significant effects in decreasing depressive symptoms but not regarding sleep compared to placebo. Regarding the use of melatonin agonist ramelteon as a prophylactic treatment, melatonin did not demonstrate preventing relapses in a recent and large RCT of 477 patients with BD type I in remission (45). These results are not in line with a previous RCT from Norris and collaborators who reported an efficacy of ramelteon 8 mg when administered over 24 weeks on the reduction of relapse rates in 83 patients with remitted BD and insomnia symptoms, for both depressive or manic episodes (46). These results may emphasize that melatonin could be more efficient in patients with insomnia or circadian rhythm disorders.

Benzodiazepines are frequently prescribed long-term for the treatment of insomnia in patients with severe mental illness. This prescribing practice is problematic because of well-described side effects including risk of dependence. Baandrup et al. (47) examined the efficacy of PR melatonin 2 mg on objective and subjective sleep quality during benzodiazepine discontinuation and whether sleep variables were associated with benzodiazepine withdrawal in patients with schizophrenia, schizoaffective disorder, or bipolar disorder and long-term use of benzodiazepines in combination with antipsychotics. Authors reported data from a subsample of 23 patients undergoing sleep recordings and 55 patients participating in subjective sleep quality ratings. PR melatonin 2 mg significantly improved self-reported sleep quality, and benzodiazepine discontinuation was not associated with rebound insomnia in these patients.

In unipolar depression, Dolberg et al. (48) conducted a randomized study assessing the effect of 5–10 mg of PR melatonin combined with fluoxetine 20 mg and observed an efficacy in the treatment of insomnia symptoms. This was confirmed by a later study from Serfaty et al. (49) who tested in a double-blind RCT using 6 mg of PR melatonin as an adjuvant to antidepressant treatment in 31 patients with insomnia and showed a decrease in depressive symptoms and in sleep disturbances. Fava et al. (50) carried out a double-blind RCT and observed a significant antidepressant effect of the combined buspirone at 15 mg and PR melatonin at 3 mg.

In SAD, IR melatonin has been combined with light therapy in several trials and an antidepressant role has been suggested with improvement in sleep parameters (11). For SAD, French recommendations suggested to optimize antidepressant effect by administering non-soporific doses of IR melatonin in the afternoon/evening or in the morning according to the phase shift (11). Indeed, Lewy and collaborators compared 44 patients with SAD treated with IR melatonin administered through capsules with each capsule containing 0.225 or 0.3 mg/day, to 24 individuals treated with placebo, for 3 weeks. Some participants received melatonin capsules in relation to their phase and in order to correct a phase advance or delay and others at an inappropriate time. An improvement in depressive symptoms was observed in both groups, with a larger effect when melatonin was optimally administered according to phase shift (51). Thus, IR melatonin could have an antidepressant effect on SAD associated with a significant phase shift if it is administered according to the advanced or delayed phase profile. Most patients presenting a clinical phase delay, a better response to IR melatonin is expected when administered 2–6 h before bedtime (11).

In a study aimed to explore the effects of PR melatonin at 2 mg on sleep, morning awakening and well-being in 58 healthy adults exhibiting subsyndromal SAD and/or the negative or positive type of weather-associated syndrome were randomized to either 2 mg of PR melatonin or placebo 1–2 h before desired bedtime over 3 weeks. The study revealed that melatonin administration significantly improved the quality of sleep and vitality in the subjects with SAD (52).

Regarding tolerance in patients with mood disorders, melatonin seems overall well-tolerated Most frequent adverse events are headache, dizziness, nausea, and no serious adverse events have been reported (11, 53, 54).

In conclusion, for unipolar/bipolar depression, melatonin may be recommended as an adjuvant treatment of insomnia symptoms and circadian rhythm disturbances while there is no clear evidence regarding an anti-depressive effect with the exception of SAD. Regarding the melatonin formula, PR at 2–10 mg should be preferred for insomnia treatment in mood disorders. Higher doses of melatonin might be needed in mood disorders compared to patients with insomnia disorder in relation to polymorphisms of circadian clock genes or to mutations in melatonin pathway-related genes and receptors which characterized patients with mood disorders.

In absence of further well-conducted trials, it is recommended to treat insomnia comorbid to mood disorders according to international guidelines. IR formulations have not been recommended for the treatment of adult insomnia, and doses lower than 2 mg were shown to be ineffective in the treatment of insomnia (9). The timing of administration of PR melatonin at 2–10 mg for insomnia should take into account the chronotype of the patients and the pharmacokinetics of the drug.

Treatment of irregular rhythms or delayed sleep phase should follow international indications by using melatonin ≤ 1 mg (11, 54); most patients with mood disorders present a clinical phase delay and respond to IR melatonin administered 2–6 h before bedtime. The timing of administration should be ideally calculating with the DLMO or with a chronotype questionnaire like the Morningness-Eveningness Questionnaire (MEQ) (55) which is considered as the gold standard measure of chronotype and has been translated into several languages. Regarding the treatment of SAD, IR melatonin should be used, preferentially at chronobiotic doses (<1 mg) and should be administered according to the advanced or delayed phase profile (11).

Melatonin at 2 mg PR may be useful during benzodiazepine or hypnotics discontinuation and may improve self-reported sleep quality during benzodiazepine withdrawal (15, 47).

### Recommendations for Melatonin Use in Mood Disorders

**Insomnia**

1) The administration of PR melatonin at 2–10 mg, 1–2 h before bedtime, should be used in the treatment of insomnia symptoms or comorbid insomnia in mood disorders.
2) The administration of PR melatonin at 2 mg may be useful during sedative-hypnotics discontinuation in mood disorders and may improve sleep quality during discontinuation
3) The chronotype of patients should be taken into account to adapt the timing of the administration
4) The administration of IR melatonin in the treatment of insomnia symptoms or comorbid insomnia in mood disorders gave uncertain results, more studies are needed for recommendation in the clinical practice

**Circadian sleep disorders**

1) Melatonin is useful in the treatment of circadian sleep disorders in mood disorders; IR melatonin ≤ 1 mg should be used and timing of administration ideally calculated with the DLMO or with a chronotype questionnaire like the MEQ.

LEGEND: PR, Prolonged Release; IR, Immediate Release; DLMO, Dim Light Melatonin Onset; MEQ, Morningness-Eveningness Questionnaire.

### Use of Exogenous Melatonin in Anxiety Disorders: Review of Evidence

No randomized controlled trials were found assessing melatonin as a treatment for insomnia or circadian rhythm disorders in anxiety disorders, including generalized anxiety disorder, obsessive-compulsive disorder, social phobia, specific phobia, or post-traumatic stress disorder. Indeed insomnia is a common comorbid condition in anxiety disorders and circadian sleep disorders are frequent. However, the use of sedative/hypnotics is controversial in these disorders (11) and preliminary data are inconclusive on an effect of melatonin in the treatment of insomnia disorder and/or sleep/wake rhythm disorder associated with anxiety and somatoform (11).

In the absence of well-conducted trials, it is recommended to treat insomnia comorbid to anxiety disorders according to international guidelines, using the formulation that is validated for insomnia disorder (8, 9). The timing of administration of PR melatonin at 2 mg for insomnia treatment should take into account the chronotype of the patients and the pharmacokinetics of the drug.

Treatment of irregular rhythms or delayed sleep phase should follow previous indications by using IR melatonin ≤ 1 mg (8, 11, 19). Patients with anxiety disorders may present a phase delay and may respond to IR melatonin administered 2–6 h before bedtime.

### Recommendation for Melatonin Use in Anxiety Disorders

**Insomnia**

1) In the absence to date of well-conducted RCTs, the administration of melatonin might be useful in the treatment of insomnia symptoms or comorbid insomnia disorder in anxiety disorders according to international guidelines for insomnia disorder treatment (>55 years 2 mg PR melatonin 1–2 h before bedtime)

**Circadian sleep disorders**

2) Melatonin might be useful in the treatment of circadian sleep disorders in anxiety disorders; IR melatonin ≤ 1 mg should be used and timing of administration ideally calculated with the DLMO or with a chronotype questionnaire like the MEQ.

LEGEND: RCT, Randomized controlled trial; PR, Prolonged Release; IR, Immediate Release; DLMO, Dim Light Melatonin Onset; MEQ, Morningness-Eveningness Questionnaire.

### Use of Exogenous Melatonin in Attention Deficit Hyperactivity Disorder in Adults: Review of Evidence

A delay in melatonin secretion was observed in adults with ADHD and insomnia, suggesting the significant contribution of disturbances in circadian rhythms to sleep disorders, especially for Delayed Sleep Phase Shift, which is very frequently associated with ADHD (11). The scientific literature on sleep in ADHD has focused largely on children and adolescents. Only one RCT has been recently published in adults in October 2020 by van Andel et al. (56) who conducted a three-armed RCT in 51 adults (18–55 y) with ADHD and DSPS receiving sleep education and 3 weeks of (1) 0.5 mg/d placebo, (2) 0.5 mg/d melatonin, or (3) 0.5 mg/d melatonin plus 30 min of 10,000 lux bright light therapy (BLT) between 07:00 and 08:00 h. Interestingly, this RCT was conducted in the participants' naturalistic home settings and melatonin or placebo administration followed individual schedules, starting 3 h before the individual DLMO, measured in saliva as marker of internal circadian rhythm, and weekly advancing by 1 h. Authors observed that these low doses of melatonin advanced the circadian rhythm and reduced self-reported ADHD symptoms (56).

Higher doses where used in child and adolescent studies and may help treating insomnia symptoms. A randomized controlled trial carried out on 105 children suffering from ADHD and chronic insomnia showed that IR melatonin, administered at 7 p.m. at doses ranging from 3 to 6 mg, provided a reduction in the severity of insomnia. In particular, an improvement of about 30 min in sleep onset latency and of about 20 min of total sleeping time an advance in the endogenous secretion of melatonin by 44 min was observed (57). The positive effects of melatonin seemed to be maintained in the long term (58). Another randomized controlled trial was conducted on 60 children with ADHD with or without insomnia, treated with IR and methylphenidate. The administration of 3–6 mg melatonin was related to an improvement in sleep onset latency and total time of sleep, but with no effect on the intensity of inattention or hyperactivity symptoms (59).

### Recommendations for Melatonin Use in Adult ADHD

**Insomnia**

1) In the absence of well-conducted trials, the administration of IR melatonin at sleep-promoting dose (2–6 mg) before bedtime could be of interest to treat insomnia symptoms associated with ADHD.

*Circadian sleep disorders*

2) In case of Delayed Shift Phase Sleep associated with ADHD, chronobiotic doses of IR melatonin ( ≤ 1 mg) should be proposed, e.g., 0.5 mg/d melatonin during 3 weeks starting 3 h before the individual DLMO and weekly advancing by 1 h to improve both the circadian rhythm disorder and ADHD symptoms.

LEGEND: IR, Immediate Release; DLMO, Dim Light Melatonin Onset; MEQ, Morningness-Eveningness Questionnaire.

### Use of Exogenous Melatonin in Autism Spectrum Disorder in Adults: Review of Evidence

A recent systematic review was conducted on sleep disturbances and circadian sleep disorders in ASD across the lifespan [for an overview see (11, 28, 60)]. The literature on the adult population is scant compared to studies on children. Although few studies (61) have investigated this topic in adult subjects, they suggest results are similar to those found compared to children. Studies have reported a high frequency of sleep disturbances and alterations of circadian sleep rhythmicity in adults with ASD such as difficulties in falling asleep, frequent and long nighttime awakenings, short sleep duration, delayed circadian phases, and evening preference such as evening chronotypes associated with ASD (62). Melatonin dysregulation, which includes delay in melatonin peak, reduction in amplitude, and alteration in genes encoding for enzymes responsible in melatonin synthesis have been hypothesized to play a role.

It has already been hypothesized that sleep disturbances and circadian sleep alterations may represent a novel therapeutic target in ASD (11, 28). According to this model, by treating sleep and circadian sleep disorders in ASD, we should contribute to an improvement in ASD symptoms. There is strong evidence about the use of melatonin in ASD, indicating its beneficial effects on sleep and autistic symptoms in children with ASD (63, 64). In this framework European drug safety approval was obtained for the pediatric version of PR melatonin at doses of 2, 5, and 10 mg (Slenyto®) which is indicated for the treatment of insomnia in children and adolescents aged 2–18 with Autism Spectrum Disorder (ASD) and/or Smith-Magenis syndrome. Where sleep hygiene measures have been insufficient, melatonin has been recommended in the treatment of sleep problems in children and adolescent with ASD (65). In children studies have shown that melatonin improves sleep disturbances showing effectiveness on sleep duration, sleep latency, and nocturnal and early morning awakenings (63, 66). Most importantly, by treating sleep and circadian sleep disorders with melatonin, an improvement in typical autistic behaviors has been shown in some studies. These positive effects on both sleep and ASD symptoms should probably be also observed in adults and warrant specific studies (11, 28, 64).

To date only one study has been conducted in 6 adults with sleep disturbances and ASD using IR melatonin at doses between 3 and 9 mg. These data confirm the results of studies investigating melatonin in children and adolescents about the effectiveness of melatonin in treating sleep disorders in adults with ASD (61). Indeed, further studies are needed and in absence of well-conducted trials in adults it might be useful to transpose available data from children about the administration of melatonin for sleep problems in adults with ASD.

### Recommendations for Melatonin Use in Adults With ASD

1) In the absence to date of well-conducted trials in adults, the administration of PR melatonin at 2–5 mg 1–2 h before bedtime could be useful in the treatment of insomnia in adults with ASD, as an extrapolation from solid available data in children.

LEGEND: PR, Prolonged Release; IR, Immediate Release; DLMO, Dim Light Melatonin Onset; MEQ, Morningness-Eveningness Questionnaire.

### Use of Exogenous Melatonin in Delirium: Review of Evidence

Delirium is a transient change to attention and cognition that develops over a short period, is fluctuating in nature, and commonly involves disruption of the sleep–wake cycle (67). It is a common condition affecting at least 10% of older patients at the time of admission to hospital and between 14 and 56% during hospitalization [for a review (26)].

Management of agitation is often a challenging aspect of delirium and is under circadian control such as the sleep-wake cycle and motor activity levels. Disturbances of the circadian sleep–wake cycle represent one of the core features of delirium, leading to the hypothesis that the neurotransmitter melatonin and changes in its metabolism may be involved in the pathogenesis of delirium. Therefore, attention has focused upon the possible role of the circadian timing system in the pathophysiology of delirium (26).

Pharmacological interventions to manage delirium include psychotropic medications such as antipsychotics and benzodiazepines. Such medications are commonly used, despite the results of a recent systematic review not supporting their use in the treatment of delirium in older hospitalized adults (26); in particular, antipsychotics may induce serious cerebrovascular adverse effects and greater mortality, particularly among patients with dementia. These effects led the US Food and Drug Administration to issue a serious warning against their use. In addition, benzodiazepines are still frequently used to treat delirium, despite their being known to possibly elicit or aggravate delirium. Both of these drugs classes are associated with an increased risk of substantial harm such as over sedation and falls and may prolong delirium duration (26, 68–71).

Four papers on systematic reviews and meta-analyses have been conducted in the last 3 years about the role of melatonin in the management of insomnia and circadian sleep disorders during delirium and in its prevention (68–71). Data support the use of PR melatonin at 2 mg 2–3 h before bedtime or IR melatonin at 3–5 mg at bed time in the management of sleep and circadian disorders, behavioral and cognitive components of delirium and may reduce the incidence of delirium in an at risk population (68–71).

### Recommendations for Melatonin Use in Delirium

**Insomnia and circadian sleep disorders**

1) The administration of PR melatonin at 2 mg or IR melatonin at 2–5 mg might be useful in the treatment of insomnia and circadian rhythm disturbances related to delirium, but consensus was uncertain, more studies are needed for recommendation in the clinical practice.
2) The administration of PR melatonin at 2 mg or 3–5 mg IR before bedtime might prevent the incidence of delirium in at risk population, but consensus was uncertain, more studies are needed for recommendation in the clinical practice.

LEGEND: PR, Prolonged Release; IR, Immediate Release; DLMO, Dim Light Melatonin Onset; MEQ, Morningness-Eveningness Questionnaire.

### Use of Exogenous Melatonin in Eating Disorder: Review of Evidence

No Randomized Controlled Trials were found assessing melatonin as a treatment for insomnia or circadian sleep disorders in eating disorders. Nevertheless, insomnia is a frequently comorbid condition in eating disorders. Insomnia is related to an increased risk of eating disorders and obesity, and vice versa eating disorders are related to more disrupted sleep. Insomnia is also linked to poorer treatment outcomes for eating disorders (72). Sleep and circadian disruptions were also confirmed to be prevalent features in eating disorders and linked to poorer treatment outcomes, but the literature on the topic as well as on attempts to address adjunctive treatment strategies is still scarce (29). Evening chronotype and delayed sleep phases are the most frequent circadian disturbances observed in eating disorders (29).

In absence of well-conducted trials, it should be useful to treat insomnia comorbid associated to eating disorders according to international guidelines, using the formulation that is validated for insomnia (8, 9). The timing of administration of PR melatonin at 2 mg before bedtime for insomnia treatment should take into account the chronotype of the patients and the pharmacokinetics of the drug.

Treatment of irregular rhythms or delayed sleep phase should follow previous indications by using IR melatonin ≤ 1 mg (4, 8).

### Recommendations for Melatonin Use in Eating Disorders

**Insomnia**

1) In the absence to date of well-conducted trials, the administration of melatonin might be useful in the treatment of insomnia symptoms or comorbid insomnia in eating disorders according to international guidelines for insomnia treatment (PR melatonin at 2 mg, 1–2 h before bedtime), but consensus was uncertain, more studies are needed for recommendation in the clinical practice

**Circadian sleep disorders**

1) Melatonin might be useful in the treatment of circadian sleep disorders in eating disorders; IR melatonin ≤ 1 mg should be used and timing of administration ideally calculated with the DLMO or with a chronotype questionnaire like the MEQ, but consensus was uncertain, more studies are needed for recommendation in the clinical practice

LEGEND: PR, Prolonged Release; IR, Immediate Release; DLMO, Dim Light Melatonin Onset, MEQ, Morningness-Eveningness Questionnaire.

### Use of Exogenous Melatonin in Neurocognitive Disorders: Review of Evidence

Neurocognitive disorders extend beyond cognitive function to involve key physiological processes, including sleep. The incidence of sleep disturbances in neurocognitive disorders ranges from 70 to 90%. Neurodegeneration is accompanied by sleep difficulties due to reduction of amplitude and phase changes of circadian rhythms such as that of melatonin secretion, as well as the disturbing influences of neurodegenerative processes on sleep. The relation between sleep and neurodegenerative diseases is bidirectional and several studies have shown that sleep disruption is a major contributor to neuropathology (30, 73–76). Alzheimer disease (AD) patients with disturbed sleep-wake rhythms did not only exhibit reduced amounts of melatonin secretions, but also a higher degree of irregularities in the melatonin pattern, such as variations in phasing of the peak. An emerging symptom of this circadian disruption is “sundowning,” a chronobiological phenomenon observed in patients with AD along with sleep and wakefulness disorder. “Sundowning” is characterized by symptoms appearing in the late afternoon or early evening, which include reduced ability to maintain attention to external stimuli, disorganized speech, and thinking, a variety of motor disturbances including agitation, wandering, and repetitious physical behaviors with emotional disturbances (30, 73–76).

The effectiveness of melatonin to improve sleep quality and total sleep time in AD and Mild Cognitive Impairment (MCI) or to improve sleep quality in Parkinson disease (PD) has already been shown in two meta-analyses and in different recent overviews (30, 76–79). In addition, chronotherapeutic interventions such as timed administration of IR melatonin has been shown to relieve sundowning and agitated behaviors.

The amplitude of the melatonin rhythm is decreased in PD patients, mainly in those exhibiting excessive daytime sleepiness. Thus, a chronobiological approach to improve circadian function, could serve as an adjuvant therapy for the non-motor manifestations of PD (30, 76). An association between motor fluctuations in PD and diurnal variation in circulating melatonin levels was shown due to potential interactions of melatonin with monoamines in the striatal complex (30, 76, 78, 79). Different doses have been used in the treatment of sleep disturbances in AD and MCI; they ranged from 3 to 9 mg of IR melatonin administered before the bedtime, 3–6 mg have been used more frequently for AD and MCI, and 2 mg PR melatonin 2 h before the bedtime. These administrations of melatonin have been shown to improve sleep quality, total sleep time, and circadian sleep regulation, and to also improve sundowning, cognitive deterioration, and behavioral disorders related to AD (30, 75, 80). For PD, 3–5 mg of IR and 2 mg PR melatonin have been shown to improve sleep quality in PD patients. However, non-motor symptoms like REM Behavior Disorder (RBD) (characterized by the occurrence of vivid, intense, and violent movements during REM sleep) precede the onset of PD for years and are indicators of worse prognosis. Daily administration of 3–12 mg of IR melatonin at bedtime or 2 mg PR melatonin has been shown to be effective in the treatment of RBD (30, 74, 75, 81).

The use of melatonin in the treatment of sleep disturbances in AD, MCI, and PD is of importance because both soporific and chronobiotic properties could be useful in insomnia and circadian alterations of these neurocognitive disorders. Melatonin might be particularly useful in these population also because of its antioxidative and neuroprotective actions; as such, different mechanisms have been hypothesized through which melatonin may halt AD and PD progression (30). At least, melatonin has several advantages over other compounds that may be used to control sleep problems, as concerns remain about the use of antipsychotic or sedative medications regarding side effects particularly next-day drowsiness, concentration and memory, and restlessness of these compounds. As already discussed, PR melatonin at 2 mg is recommended in the treatment of insomnia in healthy aging (8, 9) hence 2 mg of PR or 3–6 mg of IR melatonin should be useful in the treatment of sleep disturbances in Neurocognitive Disorder. It should be important also for improving other symptoms, which show circadian variability such as sundowning, cognitive deterioration, behavioral disorders, or other sleep problems such as RBD.

### Recommendations for Melatonin Use in Neurocognitive Disorders

**Insomnia and circadian sleep disorders**

1) The administration of PR melatonin at 2 mg might be useful 1–2 h before bedtime in the treatment of insomnia and circadian sleep disorders in Neurocognitive Disorders
2) The administration of IR melatonin 2–6 mg at bedtime might be useful in the treatment of insomnia and circadian sleep disorders in Neurocognitive Disorders
3) The use of melatonin in Neurocognitive Disorders may contribute to improve symptoms such as sundowning and behavioral disorders
4) The administration of PR melatonin at 2 mg may be particularly useful in patients with Parkinson Disease and may contribute to improve sleep quality and other sleep disturbances including Rem Behavioral Disorder (RBD)

LEGEND: PR, Prolonged Release; IR, Immediate Release.

### Use of Exogenous Melatonin in Substances Use Disorders: Review of Evidence

The ability of melatonin to mitigate different aspects of addiction neurobiology has been examined extensively in animal studies. Studies have reported the efficacy of melatonin supplementation in the control of drug-seeking behavior, opiate withdrawal/relapse, behavioral sensitization, regulation of the sleep, and or circadian rhythm disorders, neuroplasticity in brain areas linked to reward and emotion regulation (27, 82).

Treatment options for insomnia symptoms and disorders in substance addiction are limited, largely because traditional hypnotics that target benzodiazepine receptors are associated with abuse potential, withdrawal effects, and the potential for overdose (19, 27, 82). Melatonin supplementation has been found particularly valuable in the management of sleep and circadian rhythm disorders in these patients, but studies are limited to date.

A double-blind cross-over control study conducted in 80 patients enrolled at a community methadone maintenance clinic underwent to a benzodiazepine (BZD) withdrawal program and assessed melatonin's ability in mitigating sleep difficulties associated with BZD withdrawal. In the study, 5 mg of IR melatonin per day was administered vs. placebo, and while melatonin did not increase the likelihood of BZD discontinuation, it improved sleep quality, especially in subjects who continued to use BDZ (83).

The use of melatonin in the treatment of sleep disturbances in patients with alcohol use disorder (AUD) has been studied in one recent pilot RCT. Gendy et al. (84) studied the effect of melatonin 5 mg of IR melatonin vs. placebo on sleep disturbances in 60 treatment- seeking AUD subjects over 4 weeks. The quality of sleep significantly improved at the end of the treatment period. Nevertheless, there was no significant drug effect. Further analyses of mood showed a significant time effect where anxiety and depression scores decreased significantly after 4 weeks. In summary, the use of melatonin in sleep disorders related to AUD has good potentiality but need further studies, including the examination of higher doses.

In the absence of further trials, melatonin might be useful in the treatment of insomnia and circadian sleep disorders related to substances use disorders following international guidelines of insomnia and circadian sleep disorders treatment (8, 9, 11, 19). PR melatonin at 2 mg may be useful during benzodiazepine discontinuation and may improve sleep quality during benzodiazepine withdrawal.

### Recommendations for Melatonin Use in Substance Use Disorders

**Insomnia**

1) Melatonin might be useful in the treatment of insomnia symptoms or insomnia comorbid related to substances use disorders according to international guidelines for insomnia treatment, using PR melatonin at 2 mg 1–2 h before bedtime if >55 years old.
2) The administration of PR melatonin at 2 mg, 1–2 h before bedtime, may be useful during sedative-hypnotics discontinuation and may improve sleep quality during discontinuation

**Circadian sleep disorders**

3) Melatonin might be useful in the treatment of circadian sleep disorders in Substances Use Disorders; IR melatonin ≤ 1 mg should be used and timing of administration ideally calculated with the DLMO or with a chronotype questionnaire like the MEQ.

LEGEND: PR, Prolonged Release; IR, Immediate Release; DLMO, Dim Light Melatonin Onset; MEQ, Morningness-Eveningness Questionnaire.

### Use of Exogenous Melatonin in Schizophrenia: Review of Evidence

Treatment of sleep disturbances in schizophrenia is of particular importance because they are very prevalent. In addition, concerns remain about the use of antipsychotic or sedative medications regarding side effects, particularly next-day drowsiness, poor attention, concentration and memory, tiredness, and restlessness (85). Therefore, the use of melatonin with its positive safety profile has gained increasing consideration (86, 87). Several RCTs were found assessing melatonin in schizophrenia across four indications such as treating (i) insomnia complaints, (ii) benzodiazepine discontinuation, (iii) delayed dyskinesia, and (iv) metabolic syndrome (11).

In the following studies on chronic insomnia disorders, the impact of melatonin was studied on insomnia complaints comorbid with schizophrenia. There are two randomized controlled trials conducted on 9 and 14 subjects, carried out by the same group showed an improvement in sleep among adults with schizophrenia using 2 mg of PR melatonin in the evening at bedtime (88, 89). Authors showed a significant improvement in sleep efficacy, in sleep onset latency and an increase in the total sleep time. On the basis of polysomnographic data acquired on two consecutive nights, the same group showed an impact on the first-night effect thus suggesting that melatonin can enhance schizophrenia patients' alertness and responsiveness in unfamiliar surroundings (88). Furthermore, there was a randomized controlled trial conducted on 40 subjects with schizophrenia and demonstrating that 3 mg of IR melatonin or more with dose ranging from 3 to 12 mg, had a greater effect than placebo in the treatment of subjective insomnia symptoms among adults with schizophrenia (90).

In another study conducted in 14 schizophrenic patients with insomnia symptoms six were treated with PR melatonin at 2 mg, and the other subjects or with cognitive behavioral therapy or with standard sedative hypnotic treatment. Patients treated with melatonin showed an improvement in sleep quality and sleep routines (85).

In addition, melatonin was used as an adjunct treatment to discontinuation from benzodiazepines in a randomized controlled trial in 76 patients with schizophrenia (90). PR melatonin at 2 mg improved complaints of insomnia during benzodiazepine discontinuation in those patients (47).

Current data on insomnia treatment in schizophrenia might be in line with international guidelines of insomnia treatment, and the PR melatonin formulation at 2 mg has demonstrated efficacy in improving these sleep symptoms in schizophrenic patients. Otherwise, 3–12 mg of IR melatonin has been also used to improve sleep quality in schizophrenia. Treatment of irregular rhythms or DSPS should follow international indications by using IR melatonin ≤ 1 mg (8, 11, 19); patients with schizophrenia may present a phase delay and may respond to IR melatonin administered 2–6 h before bedtime. In addition, PR melatonin at 2 mg may be useful during benzodiazepine discontinuation in schizophrenic patients and may improve sleep quality during hypnotic discontinuation.

### Recommendations for Melatonin Use in Schizophrenia

**Insomnia**

1) Melatonin might be useful in the treatment of insomnia symptoms or comorbid insomnia in schizophrenia; the administration of PR melatonin at 2 mg, 1–2 h before bedtime, could be used in schizophrenia
2) The administration of PR melatonin at 2 mg, 1–2 h before bedtime, may be useful during sedative-hypnotics discontinuation and may improve sleep quality during discontinuation in schizophrenia
3) The administration of IR melatonin in the treatment of insomnia symptoms or comorbid insomnia in schizophrenia gave uncertain results, more studies are needed for recommendation in the clinical practice

**Circadian sleep disorders**

1) Melatonin might be useful in the treatment of circadian sleep disorders in schizophrenia; IR melatonin ≤ 1 mg should be used and timing of administration ideally calculated with the DLMO or with a chronotype questionnaire like the MEQ.

LEGEND: PR, Prolonged Release; IR, Immediate Release; DLMO, Dim Light Melatonin Onset; MEQ, Morningness-Eveningness Questionnaire.

## Conclusion

In the context of increasing interest for the use of melatonin in psychiatric disorders, the main aim of the present consensus paper was to conduct an extensive synthesis on the use of melatonin for the treatment of insomnia and circadian sleep problems in psychiatric disorders in order to address practical recommendations on its use in psychiatric clinical practice. The use of PR formulations starting from 2 mg as a dosage might be useful in the treatment of insomnia in psychiatric disorders, indeed higher doses might be needed in clinical practice probably due to variations/mutations in circadian clock genes or melatonin pathway-related genes in psychiatric disorders. Similarly, even if low doses of IR melatonin should be useful in treating circadian sleep disturbances in psychiatric disorders, doses may be increased to modify circadian sleep disorders in neuropsychiatric disorders. Nevertheless, more studies are needed to improve these detailed recommendations, especially regarding anxiety and eating disorders, delirium, substance abuse disorders, and adults with ASD and ADHD (91, 92). More works are also expected to better characterize responders to melatonin and to better personalize its use in patients, including chronotypes and biological rhythms, which are underexamined within existing studies. For this reason, this work would like to represent also a call to actions for the evaluation and treatment of insomnia and circadian sleep regulation in psychiatric disorders (93–97).

## Data Availability Statement

The original contributions presented in the study are included in the article/supplementary material, further inquiries can be directed to the corresponding author.

## Author Contributions

LP and PAG performed the systematic search and wrote the paper. RM, EA, MA, RB, SB, PB, J-AM, PGi, LG, RL, CM, GP, JM, AM, PP, SR, IP, LN, GB, and CS voted on recommendations and revised the paper. All authors contributed to the article and approved the submitted version.

## Conflict of Interest

The authors declare that the research was conducted in the absence of any commercial or financial relationships that could be construed as a potential conflict of interest.
